# Impairment in social interaction and hippocampal long-term potentiation at perforant pathway-dentate gyrus synapses in a prenatal valproic acid-induced rat model of autism

**DOI:** 10.1093/braincomms/fcac221

**Published:** 2022-09-05

**Authors:** Reihaneh Mohammadkhani, Reza Ghahremani, Iraj Salehi, Samaneh Safari, Seyed Asaad Karimi, Mohammad Zarei

**Affiliations:** Neurophysiology Research Center, Hamadan University of Medical Sciences, Hamadan 65178/518, Iran; Neurophysiology Research Center, Hamadan University of Medical Sciences, Hamadan 65178/518, Iran; Department of Exercise Physiology, Faculty of Sport Sciences, University of Birjand, Birjand 9717434765, Iran; Neurophysiology Research Center, Hamadan University of Medical Sciences, Hamadan 65178/518, Iran; Neurophysiology Research Center, Hamadan University of Medical Sciences, Hamadan 65178/518, Iran; Department of Neuroscience, School of Science and Advanced Technologies in Medicine, Hamadan University of Medical Sciences, Hamadan 65178/518, Iran; Neurophysiology Research Center, Hamadan University of Medical Sciences, Hamadan 65178/518, Iran; Department of Neuroscience, School of Science and Advanced Technologies in Medicine, Hamadan University of Medical Sciences, Hamadan 65178/518, Iran; Neurophysiology Research Center, Hamadan University of Medical Sciences, Hamadan 65178/518, Iran

**Keywords:** autism spectrum disorder, valproic acid, social interaction, long-term potentiation, hippocampus

## Abstract

It is well established that prenatal valproic acid exposure in rats leads to autism-like behaviours and social deficits. Long-term potentiation changes in the brain have been proposed as a potential mechanism in the development of autistic behaviour. However, there are controversies regarding the effect of *in utero* valproic acid exposure on long-term potentiation. This study examined the social interaction and long-term potentiation induction in perforant pathway-dentate gyrus synapses in male offspring of a rat model of autism induced by prenatal exposure to valproic acid. On Embryonic Day 12.5, the pregnant dams received an injection of 500 mg/kg valproic acid (intraperitoneal) to produce the autism model. The sociability test was performed between Postnatal Days 37 and 40. The offsprings were urethane-anaesthetized and placed into a stereotaxic apparatus for surgery, electrode implantation and field potential recording on Postnatal Days 45–55. In the dentate gyrus region, excitatory postsynaptic potential slope and population spike amplitude were measured. Valproic acid-exposed offspring showed significantly impaired social interaction. The birth weight in valproic acid-exposed rats was significantly lower than in control rats. The ability of dentate gyrus synapses to induce long-term potentiation was hampered by valproic acid exposure. The decreasing excitatory postsynaptic potential slope and population spike amplitude of long-term potentiation provide evidence in favour of this notion. It is widely supposed that the hippocampus plays a central role in the process of learning and memory as well as social interaction and social memory. Therefore, deficiencies in hippocampal synaptic plasticity may be responsible, at least in part, for the social interaction deficits in valproic acid-exposed rats.

## Teaser

Mohammadkhani *et al.* showed that prenatal administration of sodium valproate, an antiepileptic medication known to increase the risk of autism, leads to impaired social interaction in offspring. Sodium valproate exposure also alters synaptic plasticity in the hippocampus. Hippocampal synaptic plasticity plays an important role in social memory.

## Graphical Abstract

**Graphical abstract:**
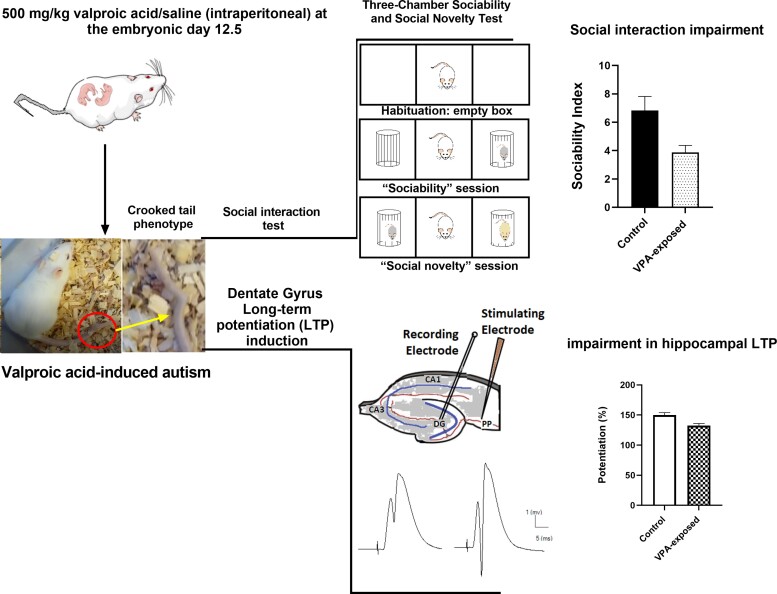

## Introduction

The prototypical neurodevelopmental disorder known as an autism spectrum disorder (ASD) is characterized by deficits in social interaction, language impairment, communication disorder, stereotyped and repetitive behaviours, limited interests and activities, and occasionally abnormal sensitivity to sound or touch, as well as aggressive behaviours.^1^ It seems that to understand the cause of neurodevelopmental disorders and design appropriate treatment strategies for them, genetic and environmental risk factors must be translated into cellular and circuitry mechanisms that underlie brain function. According to current studies, ASD is more likely to manifest in infants whose mothers took antiepileptic drugs like valproic acid (VPA) while pregnant.^2,3^ Additionally, research in rodents has demonstrated that maternal exposure to VPA increases the likelihood that offspring would develop autism, creating an animal model of ASD that closely resembles the various traits of ASD patients.^4,5^

Autism is thought to be a developmental syndrome of the hippocampus.^6^ Hippocampus is essential for the normal development of the child in syntax, semantics and pragmatics.^6^ The hippocampal formation itself is composed of several subregions, including the cornu ammonis (CA1, CA2, CA3 and CA4), the dentate gyrus (DG) and the subiculum and is important for cognitive functions and social memory.^7^

Alterations in synaptic plasticity and brain connectivity have been suggested as probable causes of autistic behaviour, and synaptic plasticity impairment occurs before the appearance of morphological alterations in ASD.^8,9^ Based on the various ASD-related pathways, it can be claimed that alterations in synaptic plasticity are involved in ASD pathology.^10^ Modifications in synaptic transmission and synaptic strength are called synaptic plasticity, which is one of the basic mechanisms involved in the process of learning and memory.^11^ Interestingly, many ASD-related mutations lead to changes in synaptic proteins that play an important role in synaptic development.^12^ It has also been reported that synaptic dysfunction and synaptopathy play a chief role in brain developmental disorders such as autism.^13,14^ Long-term depression (LTD) and long-term potentiation (LTP) are the two main classes of synaptic plasticity.^15^

Changes in LTP in the brain have been suggested as a possible cause of autistic behaviours. In animal models of ASD, several aberrant synaptic plasticity forms from several brain regions, including the hippocampus, somatosensory cortex, cerebellum, basolateral amygdala and visual cortex, have been documented.^16^ However, there are controversies regarding the effect of *in utero* VPA exposure on LTP. For instance, 2-week-old rats that were prenatally exposed to VPA demonstrated enhanced postsynaptic LTP in the neocortex.^17^ Moreover, prenatal exposure to VPA has been demonstrated to improve medial prefrontal cortex (mPFC) LTP.^18^

On the other hand, it has been shown that hippocampal LTP is attenuated in the CA1 region of the animal model of ASD^19^ and also LTP at the medial perforant path-dentate gyrus (PP-DG) synapse in the Fmr1-KO and Pten conditional knockout mouse model of autism.^20,21^ To date, little is known about the effect of *in utero* VPA exposure on hippocampal LTP at PP-DG synapses, and the impact of VPA exposure on synaptic plasticity in the VPA model of autism is unclear. Due to the existing contradictions about LTP, in this work, we aimed to investigate the social interaction and induction of hippocampal LTP in PP-DG synapses in male offspring of a rat model of ASD induced by prenatal exposure to VPA.

## Materials and methods

### The VPA rat model of autism

**Figure 1:**
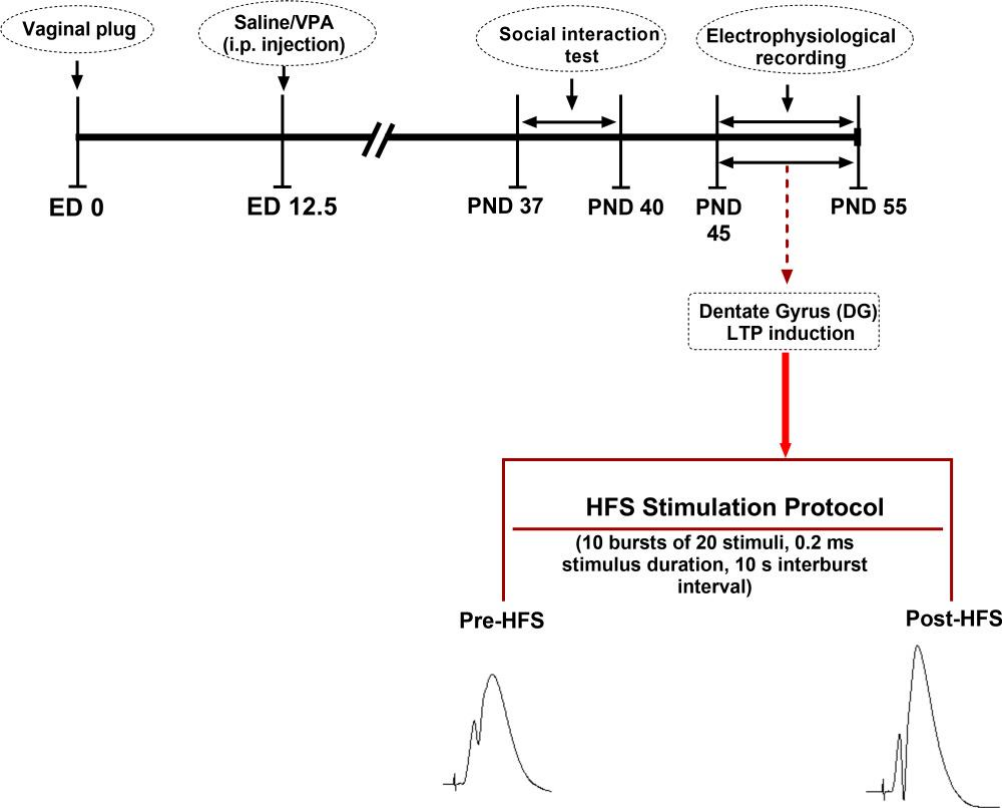
**Study design and timeline.** The first day of pregnancy was determined by the presence of sperm in vaginal smear. To induce the ASD model, the pregnant dams were injected 500 mg/kg VPA (i.p.) at the Embryonic Day 12.5. The sociability test was performed between Postnatal Days 37 and 40. Electrophysiological LTP recordings of PP-DG synapses were made between Postnatal Days 45 and 55. HFS, high-frequency stimulation.

All of the study’s experimental procedures received approval from our university’s animal study ethics committee (Code of Ethics Committee: IR.UMSHA.REC.1397.931). Additionally, the National Institutes of Health Guide for Care and Use of Laboratory Animals was followed for all experimental methods. Every effort was made to minimize suffering. For pregnancy, one sexually mature male Wistar rat was mated overnight with two female rats of the same strain. There were seven impregnated dams. The presence of a vaginal plug or sperm in the vaginal smear the following morning [on Embryonic Day 0 (E0)] was used to confirm coition. A 150 mg/ml concentration of sodium valproate (NaVPA, Sigma) was dissolved in saline (to induce a rat model of autism). VPA-dams (*n* = 7) received a single intraperitoneal (i.p.) injection of NaVPA (500 mg/kg, 3.3 ml/kg) on E12.5; whereas saline-dams (*n* = 3) received a single injection of saline as the vehicle (i.p., 3.3 ml/kg).^22^ The rats were housed in a temperature-controlled room (22 ± 2°C) under a 12-h light/dark cycle with free access to food and water. The dams were kept separately and allowed to grow their own litter. On Postnatal Days 37–55, the offspring were used for experiments. The experimental timetable is shown in Fig. 1.

### Social interaction test

Impaired social interaction is one of the most common and significant characteristics of autistic cases; as a result, the impact of VPA exposure on social behaviour was examined. The night before the test, rats were separated and housed individually. Using a box (114 cm × 51 cm × 51 cm) with three equal-length communication chambers divided by separating Perspex walls and central openings that permitted access to all chambers, the sociability test was conducted between Postnatal Days 37 and 40. In the first phase (habituation phase), the test animal was left in the empty central chamber for 5 min while both doors were left open so it could freely explore the three chambers. Then, in the second phase (sociability test), an empty wire cage (12 cm × 18 cm × 12 cm) was positioned in the centre of one side (empty cage chamber); likewise, an unfamiliar male rat of the same age and sex who had never interacted with the test rat was positioned in another small wire cage on the opposite chamber (rat cage chamber). The doors between the chambers were then opened and the test rat was free to roam all chambers for 10 min. The sociability test was repeated for 3 days, and using the Maze Router homemade software, a video-tracking system for automation of behavioural experiments, the amount of time spent in each chamber and the number of entries into each chamber were measured. In the third phase, the social preference test was carried out, and the empty cage chamber was replaced with a new, unfamiliar male rat that had never previously interacted with the test rat. The social novelty preference test was carried out to further assess the test animal’s preference to spend more time with the unfamiliar compared to the familiar rat. Each animal underwent a 10-min social novelty test, and both the number of entries and the amount of time spent in each chamber were recorded.^23^ Additionally, the social preference index (SPI) and sociability index (SI) were calculated. The SI was calculated as the ratio of time spent on Stranger 1 side over the time spent on the empty side.^24^ The SPI was determined as the ratio of time spent on the novel side over the time spent on the familiar side.

### The surgical procedure, electrophysiological recording and LTP induction

The methods applied here were done in accordance with our earlier works.^25–27^ In a nutshell, the rats were placed in a stereotaxic device for electrode implantation surgery and field potential recording between Postnatal Days 45 and 55, following urethane anaesthesia (1.5 g/kg, i.p.). The animals’ body temperature was maintained at 36.5 ± 0.5°C using a heating pad. Through tiny holes bored into the skull once the skull had been exposed, two bipolar stimulating and recording electrodes were implanted in the right cerebral hemisphere. The electrodes were constructed from Teflon-coated stainless steel (125 µm diameter, Advent Co., UK). According to the Paxinos and Watson atlas of the rat brain, the electrode placement coordinates were as follows.^25,28^ The stimulating electrode in the medial PP (Anterior-Posterior [AP]: −8.1 mm from bregma; Medial-Lateral [ML]: +4.3 mm from midline; Dorsal-Ventral [DV]: 3.2 mm from the skull surface), and the recording electrode in the DG granular cell layer (AP: −3.8 mm from bregma; ML: +2.3 mm from midline; DV: 2.7–3.2 mm from the skull surface). The electrodes were lowered from the cortex to the hippocampus extremely gently (0.2 mm/min) to reduce damage to the brain.

The input/output (I/O) curve for each animal was used to calculate the stimulus intensity for that animal. Square waves with intensities ranging from 100 to 1200 μA were applied to the PP to determine the I/O, and the evoked responses were then recorded. Baseline and high-frequency stimulation (HFS) currents were measured using the minimal intensity that, according to the I/O curve, produced the highest population spike (PS) amplitude. The 40 and 80% of the maximum intensity were used as baseline and HFS currents, respectively.

Single 0.1 ms biphasic square wave pulses at a frequency of 0.1 Hz were given by constant current isolation units (A365 WPI). The field potential recordings evoked by stimulation of the PP were recorded extracellularly in the DG region. Every 10 s, test stimuli were delivered to the PP. After recording the steady-state baseline response for 40 min, LTP was induced using the 400-Hz HFS (including10 bursts of 20 stimuli, 0.2 ms of stimulus duration and 10 s of interburst interval). To reveal any changes in fEPSP and PS, DG granular neurons were recorded at 5, 30 and 60 min after HFS. For each time point, an average of 10 consecutive recordings were made with a 10-s stimulus interval.^29–31^

For stimulations, stimulus parameters were defined in the software at the beginning and before delivery to PP, then delivered via a data acquisition board connected to a constant current isolator unit (A365 WPI, USA). The evoked responses in the DG region after passing through a preamplifier, were amplified (1000×) (Differential amplifier DAM 80 WPI, USA), and were filtered (bandpass 1 Hz–3 kHz). These responses were digitized at a sampling rate of 10 kHz and were saved on a PC for offline analysis using homemade software (eProbe, Iran).

### Measurement of evoked potentials

**Figure 2:**
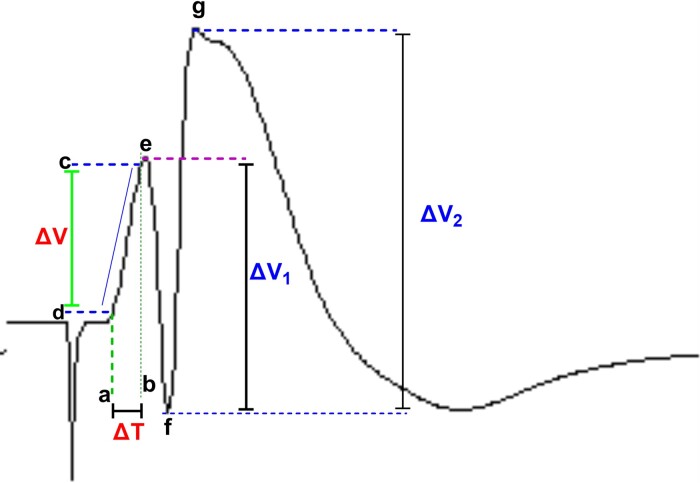
**Measurement of evoked potentials.** EPSP slope and PS amplitude were calculated according to Equations (1) and (2), respectively (see the text). Δ*V* indicatesthe potential difference, Δ*T* indicates time difference.

Two elements of the evoked recording in the DG are PS and fEPSP. In our research, we calculated the PS amplitude and fEPSP slope according to Equations (1) and (2), respectively^32^ (see Fig. 2).

(1) $$\mathrm{EPSP}=\frac{\Delta V}{\Delta T}$$

 

(2) $$\mathrm{PS}=\frac{\Delta V_{1}+\Delta T_{2}}{2}$$

where Δ*V* is the potential difference between points c and d; Δ*T* is the time difference between points a and b; Δ*V*_1_ is the potential difference between points e and f; Δ*V*_2_ is the potential difference between points f and g (see Fig. 2).

## Data and statistical analysis

For statistical analysis, GraphPad Prism^®^ 8.0.2 software (San Diego, CA, USA) was utilized. Data were presented as mean values ± standard error mean (SEM). The normality of the data was examined using the Shapiro–Wilk test. All data passed the normality test (Shapiro–Wilk test was greater than 0.05). One-way ANOVA and two-way repeated-measures ANOVA were used for multiple comparisons followed by Tukey’s and Bonferroni post-test, respectively. Two-tailed unpaired Student’s *t*-test was used when only two values were compared. LTP data were normalized to the mean value of fEPSP slope and PS amplitude recorded before the LTP induction (Equation 3).^33^  *P* values of <0.05 were considered statistically significant.

(3) $$\mathrm{LTP}=\frac{\mathrm{The}\;\mathrm{EPSP}\;\mathrm{or}\;\mathrm{PS}\;\mathrm{value}\;\mathrm{after}\;\mathrm{HFS}\;\mathrm{induction}\times 100\text{\%}}{\mathrm{The}\;\mathrm{average}\;\mathrm{EPSP}\;\mathrm{or}\;\mathrm{PS}\;\mathrm{at}\;\mathrm{baseline}}$$

### Data availability

The corresponding author will provide raw data in support of this study’s findings upon reasonable request.

## Results

### Effect of VPA exposure on birth weights

**Figure 3:**
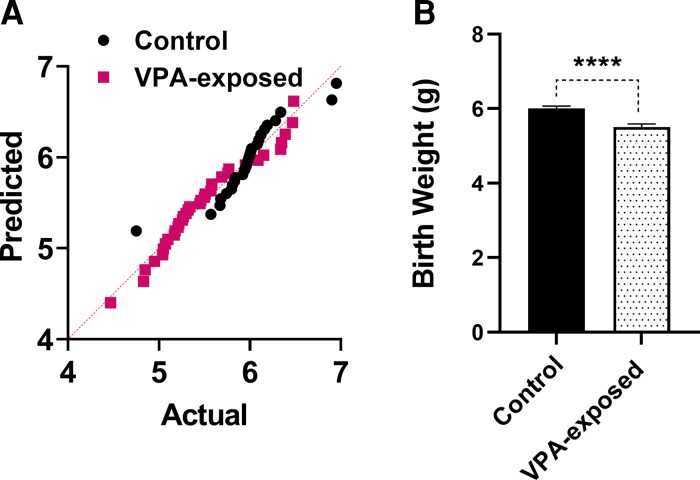
**Effect of VPA exposure on birth weights.** (**A**) Q–Q plot for distribution of data. Each point represents data for male control and VPA-exposed animals. (**B**) Birth weight was significantly decreased by VPA exposure. Bars represent mean ± SEM. ****P < 0.0001 (two-tailed unpaired Student’s *t*-test).

Given that low birth weight is an established risk factor for neurodevelopmental disorders, it is reasonable to consider it as a possible risk factor for ASD, and a lower birth weight indicates a higher risk of autistic traits.^34^ As shown in Fig. 3, VPA exposure affects offspring birth weight. The data’s normality was examined using the Shapiro–Wilk test. Fig. 3A displays the Q–Q (quantile–quantile) plot for the data distribution. The two-tailed unpaired Student’s *t*-test was employed since the data passed the normality test. Birth weight was significantly decreased by VPA exposure (*t*_65_ = 4.479, *P* < 0.0001, Fig. 3). The birth weight in VPA-exposed rats was 5.508 ± 0.08 g (*n* = 36), which was significantly lower than control rats 6.003 ± 0.06 g (*n* = 31) (Fig. 3B).

### Social interaction behaviour in rat dams prenatally exposed to VPA

**Figure 4:**
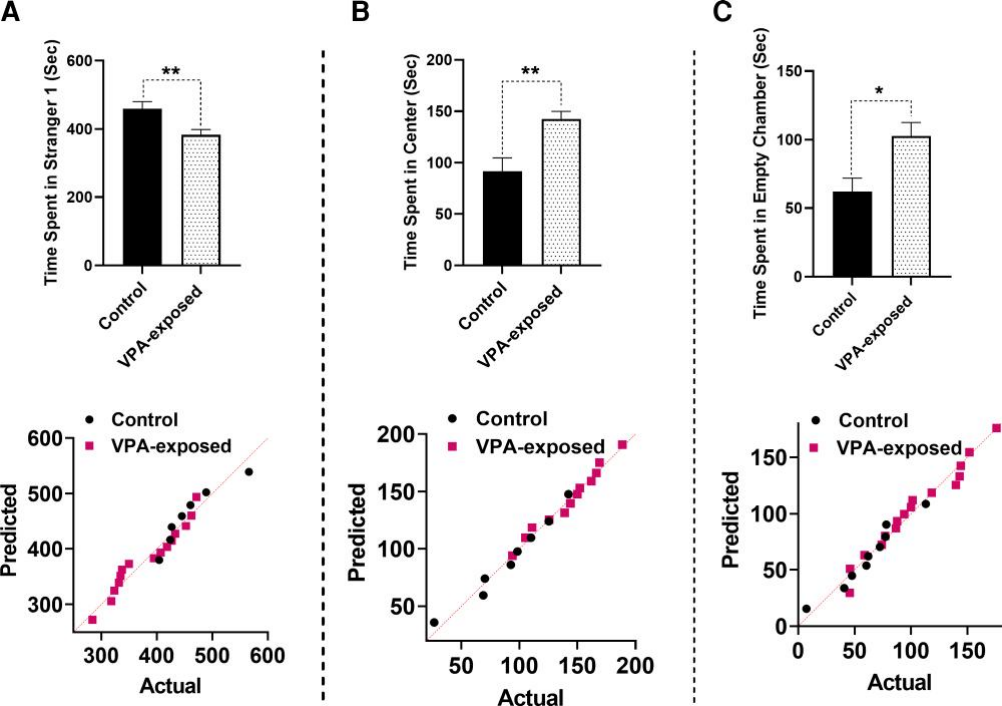
**Reduced sociability in VPA-exposed rats.** Maternal exposure to VPA caused a significant decrease in stay duration in Stranger 1 (**A**) and a significant increase in the time spent in the central (**B**) and empty chamber (**C**). The lower panels show the Q–Q plot for the distribution of the data. Each point represents data for male control and VPA-exposed animals. Bars represent mean ± SEM. *P < 0.05, **P < 0.01 (two-tailed unpaired Student’s *t*-test).

**Figure 5:**
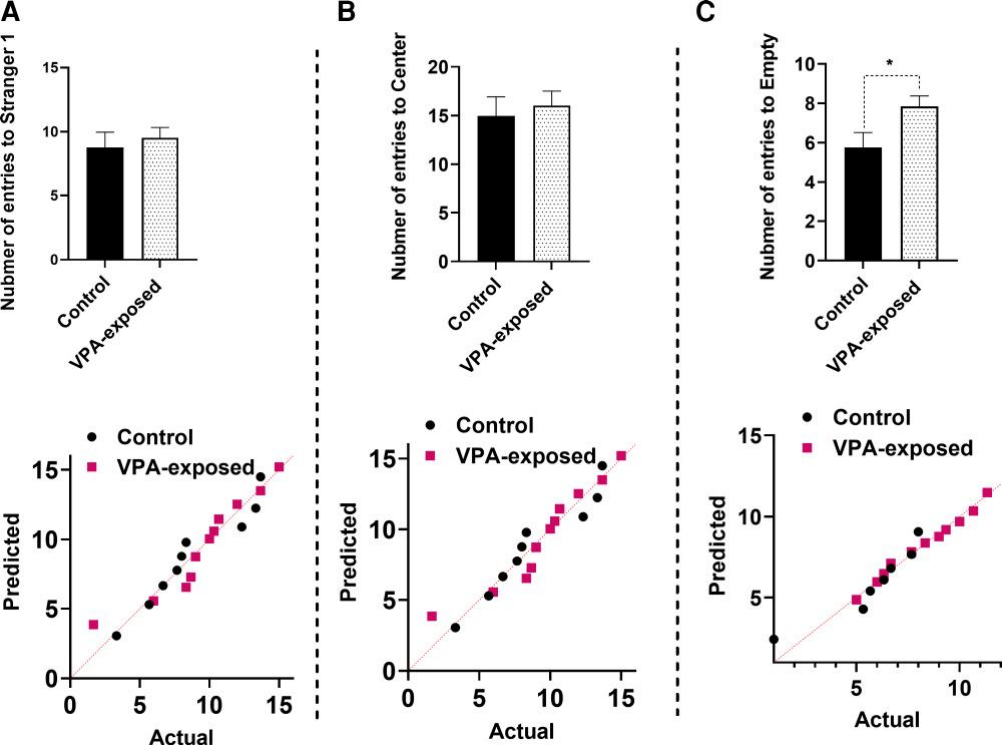
**Reduced sociability in VPA-exposed rats.** The number of entries into stranger (**A**) and centre (**B**) sides were not changed by VPA exposure. VPA exposure increased the number of entries into the empty side (**C**). The lower panels show the Q–Q plot for the distribution of the data. Each point represents data for male control and VPA-exposed animals. Bars represent mean ± SEM. *P < 0.05 (two-tailed unpaired Student’s *t*-test).

As reported previously, VPA injection caused significant alterations in the social interaction of offspring rats. In this study, offspring rats born from dams treated with VPA on E12.5 were evaluated for sociability on Postnatal Days 37–40, which demonstrated clearly impaired social interaction. All data shown in Figs 4 and 5 passed the normality test. The data’s normality was examined using the Shapiro–Wilk test. The lower panels of Figs 4 and 5 display the Q–Q plot for the distribution of the data. Our results indicated that stay duration in Stranger 1 side was reduced by VPA exposure (*t*_20_ = 2.846, *P* = 0.01, Fig. 4A). In addition, maternal exposure to VPA caused a significant increase in the time spent in the central (*t*_20_ = 2.846, *P* = 0.0018, Fig. 4B) and empty chamber (*t*_20_ = 2.702, *P* = 0.0127, Fig. 4C). In Fig. 5A and B, entries into Stranger 1 (*t*_20_ = 0.5456, *P* = 0.5908, Fig. 5A) and into the centre (*t*_20_ = 0.4334, *P* = 0.6690, Fig. 5B) side was not changed by VPA exposure. Entries into the empty side were changed by VPA exposure (*t*_20_ = 2.265, *P* = 0.0347, Fig. 5C). VPA exposure increased the number of entries into the empty side.

**Figure 6:**
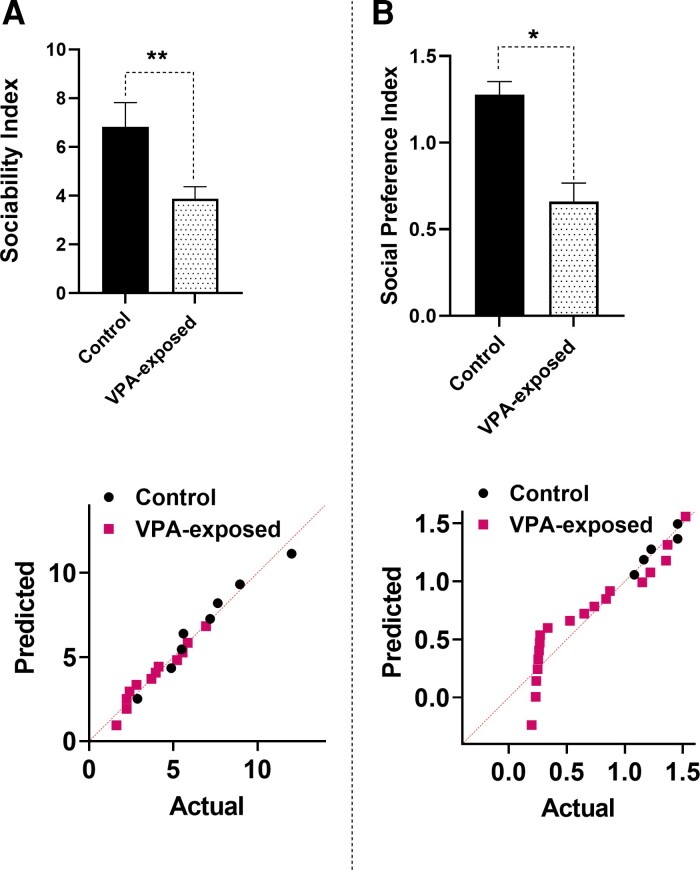
**Reduced social preference in valproic acid VPA-exposed male rats.** The sociability index (**A**) and social preference index (**B**) were significantly decreased by VPA exposure. The lower panels show the Q–Q plot for the distribution of the data. Each point represents data for male control and VPA-exposed animals. Bars represent mean ± SEM. *P < 0.05, **P < 0.01 (two-tailed unpaired Student’s *t*-test).

The results showed that the SI of autistic rats was significantly lower than those of control rats (*t*_20_ = 2.931, *P* = 0.0089, Fig. 6A). The SI was determined as the ratio of time spent on the Stranger 1 side over time spent in the empty side. The SPI was significantly decreased by VPA exposure (*P* = 0.0152, Fig. 6B). The SPI data did not pass the normality test, so we used Mann Whitney test.

### LTP at PP-DG synapses

**Figure 7:**
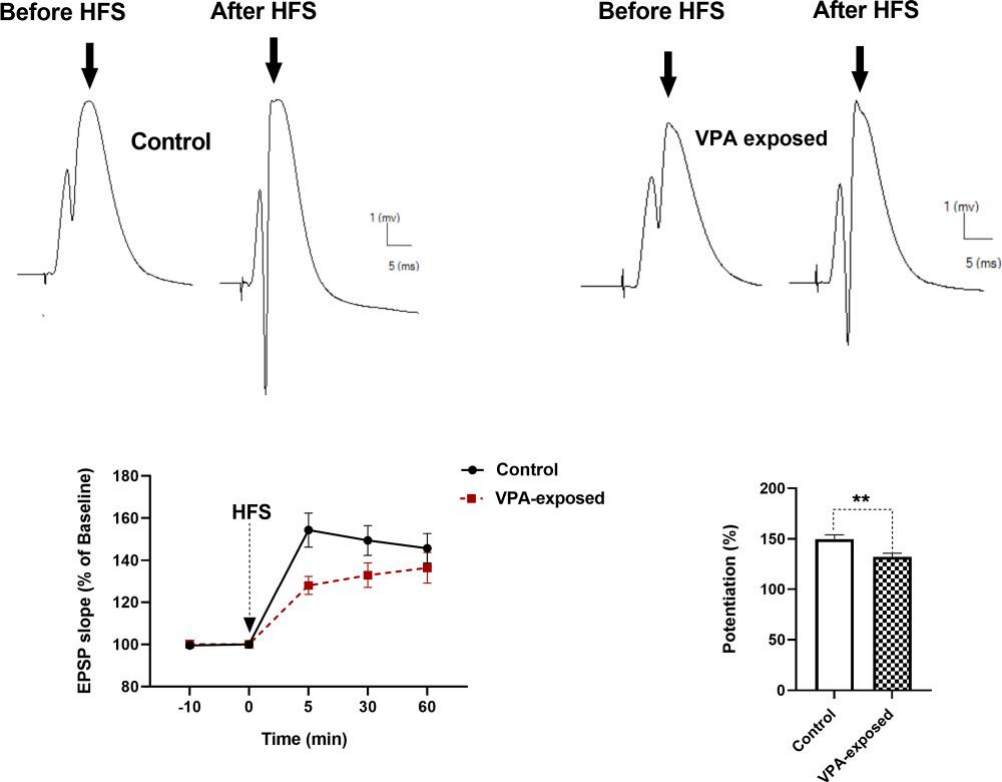
**Time-dependent changes in hippocampal responses to perforant path stimulation following an HFS.** The upper panel shows a representative example of evoked field potential in the DG recorded before and 60 min after high-frequency stimulation. VPA-exposed male rats exhibited significantly less fEPSP slope LTP than male control animals. Left lower panel shows fEPSP slope change (%) versus time following an HFS in both groups {two-way repeated-measures ANOVA; time-points effect [*F*(2.377, 61.80) = 45.57, *P* < 0.0001], VPA effect [*F*(1, 26) = 4.297, *P* = 0.0482] and interaction [*F*(3, 78) = 3.331, *P* = 0.0237]}. Each point represents data for different time point in control and VPA-exposed animals. Bar graphs show the average fEPSP slope change (%) during 60-min post-HFS. Bar graphs show the average EPSP slope change (%) during 60-min post-HFS. **P < 0.01.

When rats were anaesthetized with urethane, we looked at LTP at PP-DG synapses to gauge the variations in hippocampal synaptic plasticity. Before and after PP HFS, extracellular field potentials in the DG were recorded. Examining HFS-induced changes in the slope of the fEPSP and amplitude of the population granule cell discharge (i.e. the PS) allowed us to measure LTP. The upper panel of Fig. 7 displays an illustration of an evoked field potential in the DG that was recorded before and 60 min after HFS.

### Field EPSP LTP of DG granular neurons in offspring were affected by prenatal exposure to VPA

The magnitude of the fEPSP slope LTP differed among animals, as seen in Fig. 7. The results showed that prenatal exposure to VPA resulted in decreased fEPSP of DG granular neurons (Fig. 7). Two-way repeated-measures ANOVA revealed significant effect of time-points [*F*(2.377, 61.80) = 45.57, *P* < 0.0001], significant effect of VPA [*F*(1, 26) = 4.297, *P* = 0.0482] and a significant interaction [*F*(3, 78) = 3.331, *P* = 0.0237] in the slope of fEPSP of the granular cell of DG (Fig. 7). *Post hoc* comparisons (*P* < 0.05) indicated that VPA-exposed rats exhibited significantly less fEPSP slope LTP than control animals. The percent change in the slope of fEPSP after HFS was significantly smaller in VPA-exposed rats than in control rats (two-tailed unpaired Student’s *t*-test).

### Population spike LTP of DG granular neurons in offspring were affected by prenatal exposure to VPA

**Figure 8:**
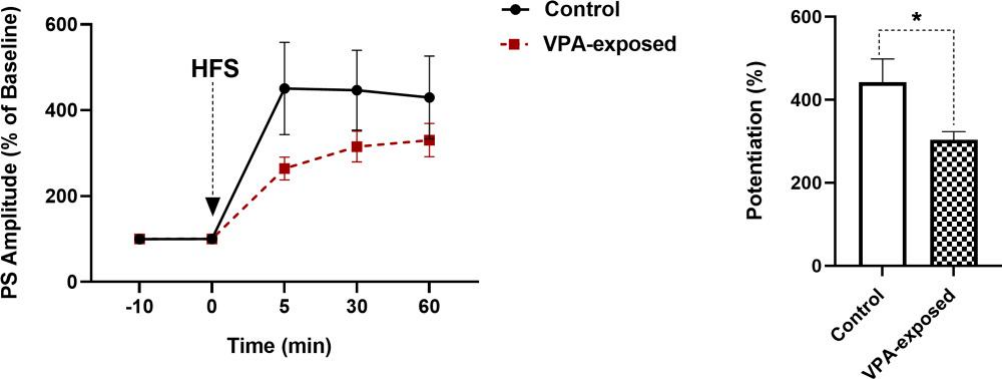
**Time-dependent changes in hippocampal responses to perforant path stimulation following an HFS.** VPA-exposed male rats exhibited significantly less PS amplitude LTP than male control animals. Left panel shows PS amplitude change (%) versus time following an HFS in both sexes of rats {two-way repeated-measures ANOVA, time-points effect [*F*(1.185, 29.63) = 26.94, *P* < 0.0001], VPA effect [*F*(1, 26) = 3.186, *P* = 0.0432] and interaction [*F*(3, 78) = 3.331, *P* = 0.0237]}. Each point represents data for different time points in control and VPA-exposed animals. Bar graphs show the average PS amplitude change (%) during 60-min post-HFS. *P < 0.05.

As shown in Fig. 8, differences in population spike LTP were also evident in the magnitude of PS potentiation. Two-way repeated-measures ANOVA revealed significant effect of time-points [*F*(1.185, 29.63) = 26.94, *P* < 0.0001], significant effect of VPA [*F*(1, 26) = 3.186, *P* = 0.0432] and a significant interaction [*F*(3, 78) = 3.331, *P* = 0.0237] in the amplitude of PS in the granular cell of DG (Fig. 8). *Post hoc* comparisons (*P* < 0.05) indicated that VPA-exposed rats exhibited significantly less PS amplitude LTP than control rats. The percent change in PS amplitude after HFS was significantly smaller in VPA-exposed rats than in control rats (two-tailed unpaired Student’s *t*-test).

## Discussion

In this work, the impact of prenatal VPA exposure on the hippocampal LTP in PP-DG synapses along with social interaction assessments was conducted on male offspring rats. A decrease in LTP and social interaction test was observed in VPA-exposed offspring. A crooked tail phenotype, which is another feature of the VPA model of autism, was also observed in our study. Moreover, the birth weight in VPA-exposed rats was lower than control rats. By this data, we confirmed the previous claim^34^ that lower birth weight indicates a higher risk of autistic traits.

The brain mechanisms underlying the impaired memory and altered social communication in autism are still unclear. The hypothesis that ASD is caused by abnormal brain growth and connectivity across regions, and synaptic plasticity deficiency is still debatable.^35^ So, the damage to LTP induction and maintenance is the cause of autism. Based on the literature, various mechanisms can be involved in the VPA-induced LTP impairment in the PP-DG synapses, which we will discuss below.

Numerous pieces of evidence indicate that DG is connected to ASD. For instance, MRI evidence from a human study showed that the area dentata of autistic men and boys were smaller than normal people.^36^ A chronic antidepressant treatment especially those that are used to treat repetitive and compulsive behaviours in autism increases neurogenesis in adult rat DG^37^ by increasing divisions of an early progenitor cell class.^38^

LTP induction requires the signalling cascades that are associated with different molecules, enzymes and protein kinases. For example, protein kinase A (PKA) activation can lead to phosphorylation of *N*-methyl-d-aspartate (NMDA) receptors as well as increased intracellular Ca^2+^ concentration and permeability, which facilitates the LTP induction.^39^ Medial PP-evoked NMDA receptor-mediated EPSCs, as well as the NMDA/AMPA ratio, have been reported to decrease in the autistic mouse model.^20^ Lee and colleagues have also shown that VPA inhibits LTP mediated by the NMDA receptor.^40^ VPA also increases inhibitory postsynaptic potential and enhances GABAergic inhibitory neurotransmission.^41^ Increased GABA_A_-mediated hyperpolarizing responses inhibit NMDA receptors and thus inhibit LTP induction.^42^ In addition, VPA could diminish repetitive neuronal firing and reduces excitability via blockade of sodium channels.^43^ These mechanisms may be involved in VPA-induced LTP damage.

Prenatal VPA has also been shown to diminish hippocampal dendritic spine density.^44^ Hippocampal LTP is linked to dendritic spine alterations^45^ and increasing dendritic spine density contributes to DG LTP.^46^

Protein kinase C (PKC) is required for LTP induction.^47^ VPA has an inhibitory impact on PKC, and VPA exposure significantly decreases PKC activity in the hippocampus.^48^

Normal hippocampal synapses that express LTP require glutamate neurotransmission. In glutamatergic and cholinergic synapses, VPA has been shown to interfere with synaptic potentiation and diminish end-plate potential.^49,50^

In contrast to the previous research, Rinaldi *et al*.^17^ found that prenatal VPA exposure enhances the expression of NR2A, NR2B and a-CaMKII in the neocortex, along with an increase in postsynaptic LTP. The conflicting findings between studies may be due to differences in the type of studied subjects, embryonic day exposure to VPA, area of LTP recording, LTP stimulation protocol, pathway-specific manner, age of offspring, study design and experiment conditions.

ASD is also associated with changes in neurogenesis and neuron migration. Synaptic plasticity could be affected by alterations in neurogenesis. In the DG of VPA-exposed offspring, neurogenesis abnormalities have been found,^51^ and maternal VPA exposure leads to neurogenesis impairment in non-human primates.^52^ Snyder *et al*.^53^ looked into the effects of neurogenesis on synaptic plasticity in DG and found that young, adult-generated granule neurons play an important role in synaptic plasticity.^53^

Presynaptic and postsynaptic proteins are also connected with ASD.^10^ Genetic studies have shown that exposure to prenatal VPA affects molecules such as neuroligins (NLGNs) and neurexins (NRXN).^54^ NLGNs and NRXNs are involved in the maturation of synapses and hippocampal LTP.^55,56^ It has been demonstrated that NLGN1 reduces NMDA receptor-mediated currents and prevents the expression of LTP at the thalamo-amygdala pathway.^57^ Similarly, both deletion and overexpression of NLGN1 were reported to impair LTP in the hippocampus.^58,59^ These mechanisms might be responsible for the VPA-induced LTP impairment in the DG region.

Changes in monoamine levels have also been observed in offspring exposed to VPA.^60^ It has been shown that monoamines can modulate the synaptic function in the DG, and selective depletion of monoamines reduces the LTP in the DG of the rat.^61^

Both increased^62^ and decreased^63^ brain-derived neurotrophic factor (BDNF) expression in the foetal brain have been reported in the VPA model of autism. The BDNF has a regulatory role in synaptic plasticity and LTP.^64^ Alterations in hippocampal BDNF may be involved in the VPA-induced LTP impairment in the DG area.

Autism is largely human-specific. Additionally, there are many restrictions and precautions in designing rodent models. The most crucial one is that animals cannot mimic all of the unique human aspects of autism. Taking together our present work showed that prenatal VPA exposure impaired hippocampal LTP. Studies on both humans and animals have shown that the hippocampus plays a significant role in social memory.^7^ So, deficiencies in hippocampal LTP may be responsible, at least in part, for the social interaction deficits in VPA-exposed rats. To fully understand the underlying mechanisms, more pharmacological, biochemical and electrophysiological studies are required.

## Abbreviations

ASD =: autism spectrum disorder
BDNF =: brain-derived neurotrophic factor
CaMKII =: Ca^2+^/calmodulin-dependent protein kinase II
DG =: dentate gyrus
EPSP =: excitatory postsynaptic potential
HFS =: high-frequency stimulation
LTP =: long-term potentiation
LTD =: long-term depression
mPFC =: medial prefrontal cortex
NMDA =: *N*-methyl-d-aspartate
NLGN =: neuroligins
NRXN =: neurexins
PP =: perforant pathway
PS =: population spike
PKC =: protein kinase C
PKA =: protein kinase A
PSD =: postsynaptic density
SI =: sociability index
SPI =: social preference index
VPA =: valproic acid
